# Flower-inducing technology facilitates speed breeding in cassava

**DOI:** 10.3389/fpls.2023.1172056

**Published:** 2023-05-22

**Authors:** Erika Paola Barinas Rodrmguez, Nelson Morante, Sandra Salazar, Peter T. Hyde, Tim L. Setter, Peter Kulakow, Johan Steven Aparicio, Xiaofei Zhang

**Affiliations:** ^1^ Universidad Nacional de Colombia, Sede Palmira, Palmira, Colombia; ^2^ Cassava Program, International Center for Tropical Agriculture (CIAT), Cali, Colombia; ^3^ Section of Soil and Crop Sciences, School of Integrative Plant Science, Cornell University, Ithaca, NY, United States; ^4^ Cassava Program, International Institute for Tropical Agriculture (IITA), Ibadan, Nigeria; ^5^ Beans Program, International Center for Tropical Agriculture (CIAT), Cali, Colombia

**Keywords:** cassava, flower-inducing, photoperiod extension, pruning, growth regulator

## Abstract

Cassava is a tropical crop that provides daily carbohydrates to more than 800 million people. New cassava cultivars with improved yield, disease resistance, and food quality are critical to end hunger and reduce poverty in the tropics. However, the progress of new cultivar development has been dragged down by difficulties obtaining flowers from desired parental plants to enable designed crosses. Inducing early flowering and increasing seed production are crucial to improving the efficiency of developing farmer-preferred cultivars. In the present study, we used breeding progenitors to evaluate the effectiveness of flower-inducing technology, including photoperiod extension, pruning, and plant growth regulators. Photoperiod extension significantly reduced the time to flowering in all 150 breeding progenitors, especially late-flowering progenitors which were reduced from 6-7 months to 3-4 months. Seed production was increased by using the combination of pruning and plant growth regulators. Combining photoperiod extension with pruning and the PGR 6-benzyladenine (synthetic cytokinin) produced significantly more fruits and seeds than only photoperiod extension and pruning. Another growth regulator, silver thiosulfate, commonly used to block the action of ethylene, did not show a significant effect on fruit or seed production when combined with pruning. The present study validated a protocol for flower induction in cassava breeding programs and discussed factors to consider in implementing the technology. By inducing early flowering and increasing seed production, the protocol helped move one step further for speed breeding in cassava.

## 1 Introduction

As a staple crop, cassava (*Manihot esculenta* Crantz) plays a crucial role in ending hunger and reducing poverty in the tropics. It provides daily energy for smallholder farmers and serves as a cash crop offering starch for industrial use (Cock, 1982; Parmar et al., 2017). As a tropical crop, cassava is only cultivated in developing countries in the Global South, with almost no production in Europe and North America (FAOSTAT, 2021). Therefore, cassava has drawn less attention than corn, wheat, and rice and attracted much less research investment. However, cassava has considerable potential for production (Adiele et al., 2020). The highest yield was reported in Tay Ninh, Vietnam, with 40-60 tons/ha, where cassava is a cash crop for starch production. With a typical dry matter content of 30%, the dry matter yield is 12-18 tons/ha, comparable with corn production in the US (FAOSTAT, 2021).

Unfortunately, worldwide, cassava yield is relatively low; for example, 7.8 tons/ha is the country average in Nigeria, 10.9 tons/ha in Colombia, and 20.3 tons/ha in Thailand in 2020 (FAOSTAT, 2021). One of the primary reasons for the higher yield in Thailand versus Columbia and Nigeria is the high planting density (20,000 vs. 10,000 plants per ha). To enable high-density planting, farmers need cultivars with erect, non-branching, plant architecture. Regrettably, flowering time and plant architecture are linked because the transition of the apical meristem to an inflorescence triggers branching. Erect genotypes with minimal branching are also late flowering and usually do not generate many seeds in crossing nurseries creating a profound dilemma for cassava breeders (Ceballos et al., 2004; Pineda et al., 2020a).

Cassava inflorescences usually have separate male and female flowers. Female flowers are at the bottom of the inflorescences and mature 1 or 2 weeks earlier than the male flowers above to avoid self-pollination. Very few hermaphrodite flowers have been observed. Bees are the primary pollinator among cassava plants in the natural environment. The inflorescences and associated flowers are derived from the shoot apical meristem. Once inflorescence development initiates, two to four buds beneath the apical meristem will develop into new branches. These branches each have shoot apical meristems, which can develop new branches and inflorescences, so there will be 1^st^ branching, 2^nd^ branching, etc. (Perera et al., 2013; Oluwasanya et al., 2021a). Thus, branching and flowering always co-occur in cassava. However, the inflorescences and flowers usually abort or do not fully develop at the 1^st^ and 2^nd^ branching (Pineda et al., 2020b). Moreover, it is common that inflorescences are mostly male flowers with only a few female flowers. Therefore, a minimal number of fruits and seeds can be harvested from each inflorescence (Halsey et al., 2008; Adeyemo et al., 2017; Hyde et al., 2020; Silva Souza et al., 2020).

Realizing the inefficiency in controlled pollination and botanical seed production in breeding programs, cassava researchers have been studying the genetics of early flowering and technologies to induce early and abundant flowering. Transgenic studies showed that overexpressing the Arabidopsis *FLOWERING LOCUS T* (FT) gene in cassava induced early flowering and branching, and increased flower quantity (Adeyemo et al., 2017; Odipio et al., 2020). Transcript analysis also provided evidence that FT genes are involved in cassava branching and flowering (Adeyemo et al., 2019; Tokunaga et al., 2022). There is still little information about the FT gene diversity in cassava populations and how to use the FT gene variation to manage the flowering habit of breeding materials. Most effort has been focused on the physiological treatments to induce early flowering, increase the inflorescence size and the number of female flowers, and reduce female flower abortion (Hyde et al., 2020; Pineda et al., 2020a; Pineda et al., 2020b; Oluwasanya et al., 2021a).

Controlled-environment studies in growth chambers have shown that cassava flower induction is favored by cool temperatures (Oluwasanya et al., 2021a; Hyde and Setter, 2022). Photoperiod extension (long days and short nights) induces early flowering in cassava (Hyde and Setter, 2022), particularly in late-branching genotypes (Pineda et al., 2020a). Photoperiod and temperature responses interact with each other, and elevated temperatures can negate the benefit of extended photoperiod (Hyde and Setter, 2022). Upon floral induction and the appearance of an inflorescence, pruning the branches beneath the apex stimulated inflorescence and floral development and reduced the flower abortion in the 1^st^ or 2^nd^ branches (Pineda et al., 2020b). The anti-ethylene growth regulator, silver thiosulfate (STS), also increased inflorescence size and flower longevity (Hyde et al., 2020). The cytokinin benzyladenine (BA) can feminize flowers and increase the number of fruits and seeds (Pineda et al., 2020b; Oluwasanya et al., 2021a). Together these techniques can be combined to decrease the time to flowering, stimulate larger inflorescences with more flowers, and increase the ratio of female to male flowers. Thus, producing seeds in a crossing nursery faster and in greater abundance.

The flower-inducing technology of photoperiod extension, new branch pruning, and growth regulator application requires high labor intensity and a cost to set up, e.g., 1,000-3,000 USD for dozens of progenitors in a national program and 10,000-20,000 USD for a couple of hundred of breeding progenitors. The effectiveness of these components and their combination has only been tested in a small set of genotypes in several studies. Large-scale validation is needed before the technology is implemented in cassava breeding practice. Thus, in the present study, our objectives were 1) to understand the response of 150 breeding progenitors to photoperiod extension; 2) to validate the effectiveness of pruning and growth regulators in fruit and seed production using 13 breeding progenitors under photoperiod extension; and 3) to ensure the feasibility of the flower inducing technology in breeding practice.

## 2 Materials and methods

### 2.1 Plant materials

The plant materials included 150 breeding progenitors from the cassava breeding program at the Alliance of Bioversity International and International Center for Tropical Agriculture (CIAT). These progenitors were selected in breeding pipelines for high dry matter content, good cooking quality, high beta-carotene content, cassava mosaic disease (CMD) resistance, cassava brown streak disease (CBSD) resistance, and whitefly resistance (**Table 1**). We planted these progenitors in the crossing nurseries in Palmira, Valle del Cauca, Colombia, in June 2020. The duration of the natural photoperiod varies from 11h 55m to 12h 20m throughout the year. The monthly rainfall ranges from 500 mm to 760 mm, on average in the past thirty years. The altitude of Palmira is 1001 meters above sea level. The temperatures at Palmira are fairly constant throughout the year, and the average daily maximum and minimum temperatures during the growth season were 30.1 ± 2.7°C and 19.2 ± 1.2°C, respectively.

**Table 1:** The 150 breeding progenitors used in the photoperiod extension experiment.

Progenitor	Group	Progenitor	Group	Progenitor	Group	Progenitor	Group	Progenitor	Group
AM1549-15	BC	COL1107	BS	Azulita	CQ	CG1141-1	DM	5G160-13	SS
GM10010-1	BC	COL144	BS	Chocoana	CQ	CM4574-7	DM	5G160-16	SS
GM10035-1	BC	COL2131	BS	COL1505*	CQ	CM4919-1*	DM	5G160-18	SS
GM1561-11	BC	COL2173	BS	COL1722*	CQ	CM6119-5*	DM	GM4682-7	SS
GM3426-5	BC	COL2182*	BS	COL2215	CQ	CM6438-14*	DM	GM4694-22	SS
GM3518-42	BC	COL40*	BS	CR138	CQ	CM9460-40	DM	GM4694-39	SS
GM3518-66	BC	ECU183	BS	CUB46	CQ	CM9912-167	DM	GM4694-4	SS
GM3667-24	BC	ECU41	BS	CUB74	CQ	CM9962-27	DM	FalseReina	SS
GM8527-12	BC	PER206	BS	GUA24	CQ	GM3594-77	DM	GM3937-67*	SS
GM8560-13	BC	PER221	BS	HMC1	CQ	GM3893-65	DM	GM4034-1*	SS
GM8956-1	BC	PER226	BS	IND135	CQ	GM579-13	DM	GM4781-2	SS
GM9404-1	BC	PER353	BS	MAL3	CQ	GM9108-5	DM	GM4883-1	SS
GM9823-1	BC	PER556	BS	MEX2	CQ	KU50	DM	GM4883-3	SS
GM9927-3	BC	PER597	BS	PAN70	CQ	SM1127-8*	DM	GM8716-1	SS
SM3536-44	BC	C19	MD	PAR98	CQ	SM1411-5	DM	SM4516-40	SS
SM3677-74	BC	C243	MD	PER183	CQ	SM2773-32	DM	SM4517-30	SS
SM4289-438	BC	C33*	MD	PER496	CQ	SM2775-4	DM	SM4517-33	SS
SM4358-15	BC	C39	MD	VEN208	CQ	SM2792-31	DM	SM4522-13	SS
SM4376-3	BC	C413	MD	VEN77	CQ	SM2828-28	DM	SM4524-28	SS
SM4388-2	BC	GM10054-1	MD	CG489-31	WF	SM2834-31	DM	SM4534-37	SS
SM4389-36	BC	GM10054-2	MD	ECU64	WF	SM3106-14	DM	SM4541-2	SS
SM4410-1	BC	GM10054-3	MD	ECU72	WF	SM3110-15	DM	SM4708-4	SS
SM4423-1	BC	GM10055-1	MD	PER415	WF	SM3134-5	DM	SM4709-12	SS
SM4471-64	BC	GM10055-2	MD	PER497	WF	SM3134-73	DM	SM4716-23	SS
SM4483-2	BC	TME3	MD			SM3137-40	DM	SM4716-27	SS
SM4483-3	BC	GM10062-1*	MD			SM3139-22	DM	SM4761-2	SS
SM4484-18	BC	GM6127-13	MD			SM3150-17	DM	SM4834-11	SS
SM4491-2	BC	GM6127-15	MD			SM3386-49	DM	SM4844-10	SS
SM4515-6	BC	GM7672-7	MD			SM3464-29	DM	SM4848-17	SS
SM4573-27	BC	GM7672-8	MD			SM3553-27	DM		
SM4574-58	BC	GM7672-9	MD			SM3559-11	DM		
		GM7673-3	MD			SMB2446-2	DM		
		GM7673-7	MD			TAI8	DM		

BC, high Beta-Carotene; BS, cassava Brown Streak virus resistance; MD, cassava Mosaic virus resistance; CQ, good Cooking Quality; WF, WhiteFly resistance; DM, high Dry Matter; SS, Specialty Starch.

All these breeding progenitors were used to demonstrate the effect of photoperiod extension, and the breeding progenitors labeled with “*” and GM971-2 were used in the pruning and plant growth regulator experiment.

### 2.2 Experimental design

We carried out two experiments in the same crossing nursery at Palmira, a photoperiod extension experiment and a photoperiod extension with plant growth regulator (PGR) experiment. The photoperiod extension experiment included all 150 breeding progenitors with two treatments, dark night (DN) with natural photoperiod, and photoperiod extension (PE) with red light illuminating the plants all night. The objective of the experiment was to test the effect of photoperiod extension on flower induction in a diverse cassava population. The 150 progenitors with one replication were randomly planted in single-row 6-plant plots with 1 meter between plants and rows. Among them, 18 progenitors were planted with two replications in each treatment to estimate heritability and thereby evaluate data quality (sufficiently small error variance such that genetic variance is substantial relative to phenotypic variation). Populations for the DN and PE treatments were planted side-by-side. The fields for the two treatments were 20 m apart. Based on years of experience at this highly uniform field site, it has been established that field spatial effects are minimal compared to progenitor effects on flowering.

**Figure 1:**
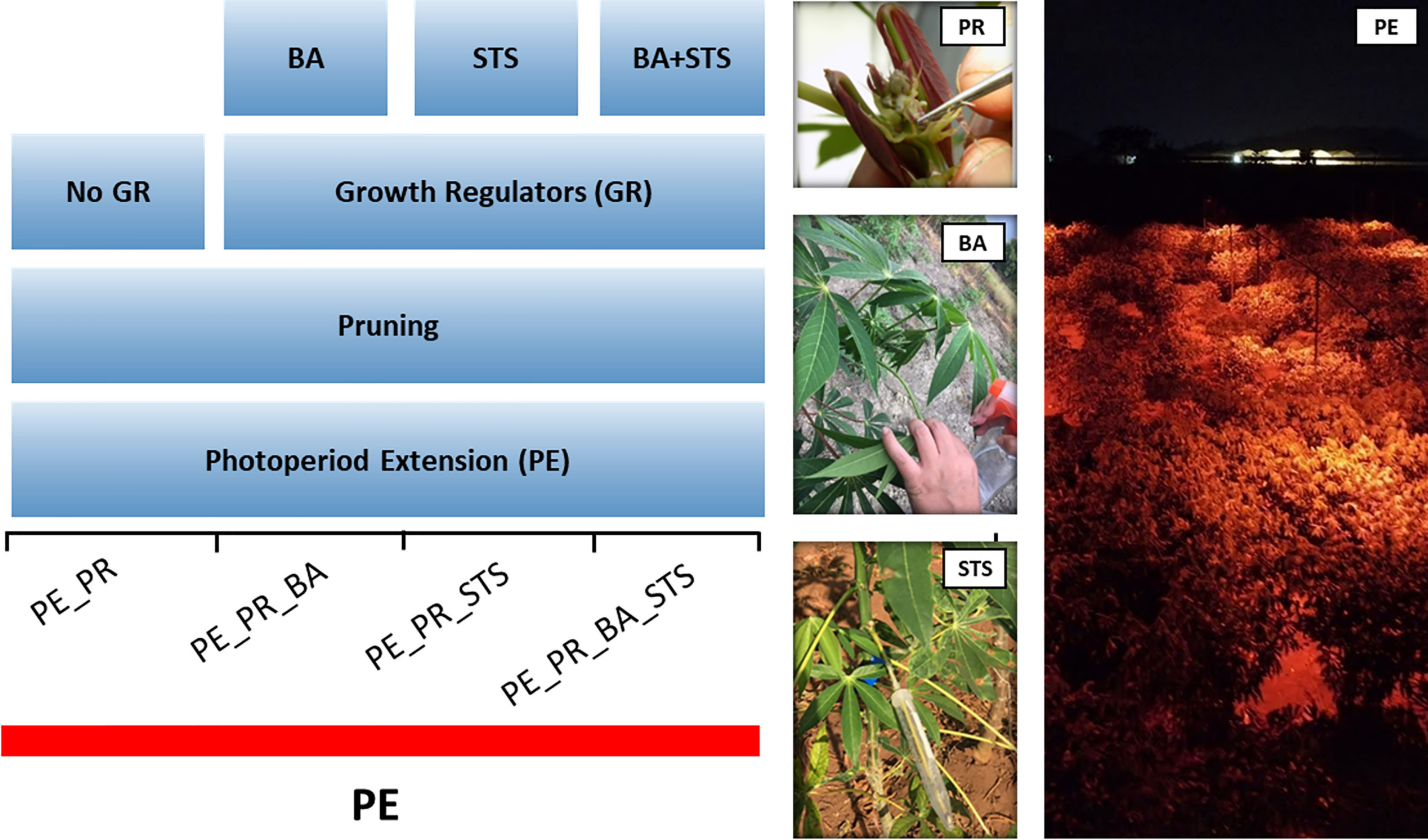
Schematic diagram of the pruning and plant growth regulator experiment, showing the four treatment combinations. The experiment involved 13 progenitors with photoperiod extension (PE) illumination for the entire experimental area. BA, 6-Benzyladenine; STS, silver thiosulfate. Photos illustrate treatment application, and PE illumination with red LEDs.

In a photoperiod extension with PGR experiment, we utilized 13 breeding progenitors with various flowering habits (**Table 1**). To obtain timely flowering, we exposed all plants to photoperiod extension, and performed four treatments, i) pruning, ii) pruning + benzyladenine (BA), iii) pruning + silver thiosulfate (STS), iv) pruning + BA + STS (**Figure 1**). Individual plants were considered the experimental unit. Breeding progenitors were planted in a grid pattern with columns of single-row plots containing 5 plants per progenitor. The four treatments were randomly assigned to the plants in each plot. There were five replications of the treatment x progenitor plots, so there were 13 × 4 × 5 (progenitors × treatments × replicates) = 260 plants in the experiment. Each plant had more than one inflorescences, and the inflorescences of the same plant had the same treatment. We called the treatment on each inflorescence as a treatment event.

### 2.3 Trial management

Field management followed the standard procedures for cassava at CIAT. A mixture of the pre-emergence herbicides Karmex (Diuron Adama, Colombia) and Dual Gold (S-metolachlor, Syngenta, Colombia) was applied 4–7 days before planting. Manual weeding was made as necessary. Plots were managed following standard procedures (Pineda et al., 2020a). Irrigation was provided *via* surface/gravity distribution, as required. Pests, particularly whiteflies (*Aleurotrachelus socialis*), were monitored weekly and controlled using pesticides (Connect Duo with active ingredient Beta-Cyfluthrin te imidacloprid from Bayer, and Starkle with active ingredient Dinotefuran from Summit Agro).

### 2.4 Photoperiod extension

Photoperiod extension was achieved by illuminating the PE field with 50-W red light-emitting diodes (LEDs; peak around 625–635 nm) at night as previously described (Pineda et al., 2020a). The 50-W LED lamps with parabolic reflectors (were hung in a fixed position of 3 m above ground in a square grid 4.5 m apart. Lights were turned on at sunset and turned off at sunrise, starting after planting. Thus, the plants were exposed to > 0.02 μmol of photons m^−2^ s^−1^ all night long.

Based on the days to the first branch under DN, we divided the population into four groups, i) the Early group (<=90 d), the 1st branching requiring equal or less than 90 days; ii) the Mid-early group (91-120 d), branching in more than 90 days but equal or less than 120 days; iii) the Mid-late group (121-150 d), branching in more than 120 days, but equal or less than 150 days, and iv) the Late group (>150 d), branching in more than 150 days.

### 2.5 Pruning

The pruning was performed when the apical shoots started transitioning to flowering, as previously described (Pineda et al., 2020b). Pruning was done when the plants were at least 80 cm tall at the first, second, or third branches. We inspected the plants twice a week starting from two months after planting, and when apical branches were first visible, removed the small branches (<5-8 mm) but did not damage the inflorescences. More than one of the 2^nd^ or 3^rd^ branches were treated for each plant.

### 2.6 Application of plant growth regulators

A 0.5 mM BA solution was sprayed until runoff to apical-region folded leaves or inflorescences immediately after pruning the lateral vegetative branches. BA was then applied weekly until the transition from flowers to fruits was observed. A 4 mM STS solution was applied through the petiole of a leaf 60 cm below the apical meristem, as previously described (Oluwasanya et al., 2021a). We cut off the leaf blade while the petiole was under water and then quickly inserted the petiole into a tube containing 2.5 ml of STS solution (4 mM). Care was taken not to kink the petiole because this will collapse the xylem vessels. A strip of Parafilm (Bemis Inc., Neenah, WI, USA) was then wrapped around the tube and petiole to hold the tube in place. Phytodamage symptoms were often observed. While a low level of symptoms indicated an adequate dose of STS had been applied, when excessive symptoms were observed, i.e., darkening and death of the leaf margins, the concentration of STS on the next applications was reduced by half for the genotype.

### 2.7 Data collection

For the 150 breeding progenitors in the photoperiod extension experiment, the dates when the 1^st^ and 2^nd^ branches were observed on each plant were recorded by visiting the plots twice a week. At ten months after planting, two representative plants per plot were measured for plant height, the height of the 1^st^ branch, and the number of branches under DN and PE.

For the 13 selected progenitors in the pruning and PGR experiment, the dates when the 1^st^ branching occurred were recorded by weekly visiting, and the height and the number of nodes to the 1^st^ branches were measured ten months after planting. To reduce the variation caused by artificial pollination, we relied on bees to pollinate the female flowers. The number of fruits per inflorescence or treatment event was recorded one month after pollination, and then the fruits were bagged. The bags were collected three months after pollination, and the seeds per inflorescence or treatment event were counted, recorded, analyzed, and reported.

### 2.8 Data analysis

In the photoperiod extension experiment with a population of 150 breeding progenitors, broad-sense heritability for the branching traits was calculated using the function H^2^ = V_g_/(V_g_ + V_e_), where V_g_ meant genetic variance; and V_e_ meant residual variance caused by field and management variation. The variance components were estimated using the lmer function in R package lme4 to run the linear mixed model with breeding progenitors as a random effect and replication as a fixed effect. The means of breeding progenitors were calculated for days of the 1^st^ branch, height of the 1^st^ branch, number of branches, and plant height. Paired t-tests were carried out using R to compare the response of different groups of progenitors to photoperiod extension. We visualized the effect of photoperiod extension of late branching progenitors using the R package, ggcharts. The correlation coefficients were calculated and visualized using the function rcorr and corrplot in R packages Hmisc and corrplot.

For the 13 breeding progenitors in the pruning and PGR experiment, there were more than six treatment events per progenitor for each treatment. The t-test was used to assess the significant difference among treatments for each progenitor. The R package ggplot2 was used to generate the figures. All the statistical analyses and result visualization were performed using R.

## 3 Results

### 3.1 Photoperiod extension induced early flowering

**Figure 2:**
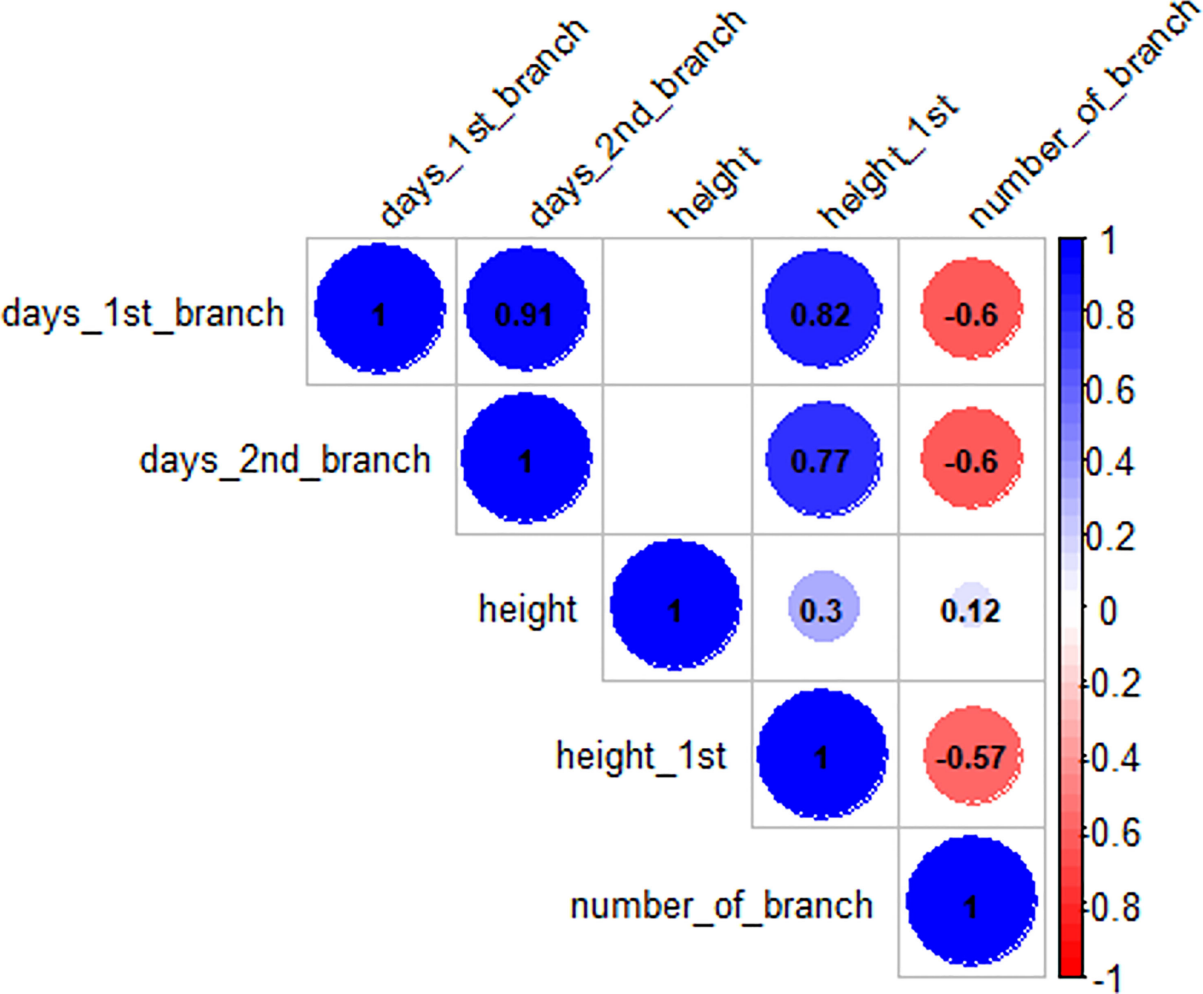
Phenotypic correlation among traits of the 150 clones under dark night and photoperiod extension. Blank or missing values showed the non-significant correlation between two variables, e.g., height and days_1st_branch.

To evaluate the data quality, we calculated the heritability using the data of 18 breeding progenitors planted in two replications under each treatment. We observed a heritability of 0.80 for the number of branches and 0.82 for the height of the 1^st^ branch in the dark night. Under photoperiod extension, we also obtained moderate heritability for the number of branches (0.44) and high heritability (0.84) for the height of the 1^st^ branch. Moreover, we consistently observed the high heritability for the days to the 1^st^ branch and 2^nd^ branch (0.85−0.94) in both the dark night and photoperiod extension. After confirming the good data quality, we performed correlation analysis and found that the days of the 1^st^ branch showed significantly positive correlation with the height of the 1^st^ branch (r = 0.82; **Figure 2**), which indicated that the easy-measure trait, height of the 1^st^ branch, can be used to indicate the earliness of branching in cassava.

**Figure 3:**
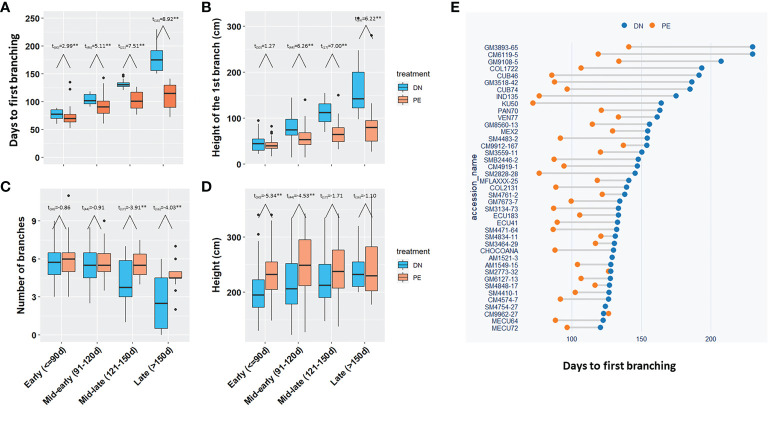
The effect of photoperiod extension on branching and height for 150 breeding progenitors. **(A)** The days to the 1^st^ branch, **(B)** The height of the 1^st^ branch, **(C)** The number of branches, and **(D)** Height of the four groups of progenitors under DN and PE; **(E)** The days to the 1^st^ branches of the breeding progenitors in Mid-late and Late groups under DN and PE. The t-test was performed to determine the significate differences between DN and PE, **for *p* value <0.01. Early (<=90d), the 1st branching requiring equal or less than 90 days; Mid-early (91-120d), branching in more than 90 days but equal or less than 120 days; Mid-late (121-150d), branching in more than 120 days, but equal or less than 150 days; Late (>150d), branching in more than 150 days. DN, dark night; PE, photoperiod extension.

Based on the days to the first branch under DN, 150 breeding progenitors were divided into four groups, i) 61 clones in the Early group; ii) 48 in the Mid-early group; iii) 24 in the Mid-late group; and iv) 17 in the Late group. All four groups required significantly (*p*<0.05) fewer days for the 1^st^ branch in photoperiod extension than in dark night (**Figures 3A, E**). The breeding progenitors with late branching (Late flowered >150 d after planting), had much earlier branching under photoperiod extension (3-4 months *vs*. 6-7 months; *p*<0.01).

Photoperiod extension also reduced height of the first branch (**Figure 3B**; *p*<0.05), which was consistent with fewer days to the 1^st^ branch. At the end of the pollination season (230 days after planting), photoperiod extension treatment in the Mid-late or Late-flowering progenitors, significantly (p<0.05) increased the number of branching events (**Figure 3C**), which reduced the population variation of the number of branches under photoperiod extension, leading to reduced heritability (0.44 vs. 0.80). The population generally had taller plants under photoperiod extension than dark night (**Figure 3D**).

In summary, for the 150 tested breeding progenitors, photoperiod extension induced early branching or flowering in cassava, and especially shortened the flowering time of late-flowering progenitors by approximately 2-3 months.

### 3.2 Pruning and growth regulators increased seed production

**Figure 4:**
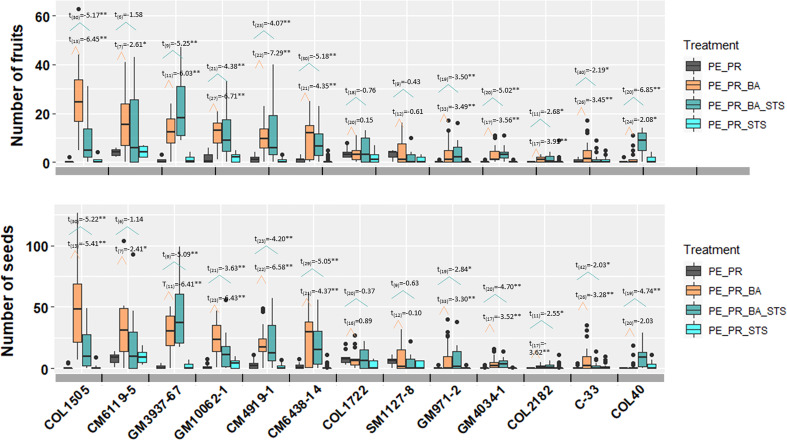
The effect four treatments on number of fruits and seeds harvested from one treatment event. The t-test results were performed and presented here. The number in subscript after t was the degree of freedom for the comparison. The broken line with orange color showed the comparison between pruning (PE_PR) and pruning and BA (PE_PR_BA). The broken line with blue color showed the comparison between pruning (PE_PR) and pruning, STS and BA (PE_PR_BA_STS). PE, photoperiod extension with red lights; DN, dark night without red lights; PR, pruning; GR, growth regulator; BA, 6-Benzylaminopurine; STS, silver thiosulfate. *for *p* value < 0.05 and **for *p* value <0.01.

In the pruning and PGR experiment with 13 breeding progenitors, each of the four treatments has more than 6 replicates per breeding progenitor, with a maximum of 42 treatment events (degree of freedom in **Figure 4**). The large number of treatment events for each breeding progenitor provided strong statistical power in comparing the effect of treatments. Among treatments that included photoperiod extension and pruning, PE_PR had the least number of seeds, and PE_PR_BA and PE_PR_BA_STS generated the largest amount of fruits and seeds (**Figure 4**). Averaged across all 13 genotypes, the number of fruits per treatment event were 0.92±1.41 in the PE_PR treatment, whereas treatments that included BA had 6.98±7.56 (PE_PR_BA) and 6.88±5.98 (PE_PR_BA_STS) fruits. Pruning + STS treatment but without BA (PE_PR_STS) was not different from pruning alone with 0.84±1.11 fruits. Seed production of the treatments showed a similar pattern to fruit production (**Figure 4**).

Among the 13 breeding progenitors, only two, COL1722 and SM1127-8 did not show a significant response in fruit and seed number to the treatments that included both pruning and growth regulators. Ten progenitors produced significantly more seeds from each PE_PR_BA event than PE_PR. For example, the erect elite progenitor, CM6119-5, produced more than 25 seeds per PE_PR_BA treatment and with more than 2-3 treatment events for each plant, thus, more than 50 seeds per plant (**Figure 4**).

## 4 Discussions

### 4.1 Considerations of implementing the flower-inducing technology

In the present study, we observed that photoperiod extension dramatically reduced the number of days to 1^st^ branch for most breeding progenitors (**Figure 3E**). These results are consistent with previous small-scale studies conducted with a limited number of genotypes (Adeyemo et al., 2019; Pineda et al., 2020a). Thus, we concluded that photoperiod extension could induce early flowering for a large quantity of diverse breeding progenitors.

We must be cautious in concluding that the effect on flowering is solely due to photoperiod extension and will be found in all environments. The elevation of Palmira is 1000 m, so the temperature is lower than that in the lowland tropics. Temperatures at Palmira compared to lowland tropics, respectively, have an average maximum of 30.1 ± 2.7 vs. 33 ± 1°C, and a minimum of 19.2 ± 1.2 vs. 23 ± 0.5°C. Studies of cassava under controlled-environment conditions of growth chambers have indicated that photoperiod and temperature interact such that the strongest flower induction is with long-day photoperiods and cool temperatures flowering (Adeyemo et al., 2019; Oluwasanya et al., 2021a; Oluwasanya et al., 2021b; Hyde and Setter, 2022). High temperatures negate the benefit of extended photoperiod (Hyde and Setter, 2022). Thus, breeding programs need to find a location with a relatively low temperature, e.g., a maximum of ≤30°C and a minimum of >15°C, to have good flowering in crossing nurseries.

Pruning the young branches below the newly developed inflorescence increases the size of the inflorescence on the first tier of branching, which is usually aborted, and results in substantially earlier and more flowers per plant (Pineda et al., 2020b). The timing for pruning is critical and must be done when the size of new branches next to the young inflorescence is less than 5-8mm, whereas pruning, when the branches are longer than 5-8mm, does not increase the size of inflorescences (Pineda et al., 2020b). Thus, the pollination team must visit the crossing nursery at least twice weekly. However, the present study shows evidence that fruit and seed production was not substantially improved by pruning unless it is accompanied by BA application (**Figure 4**). This is because, while pruning and STS increase the number of total flowers, typically ~90% of them are male and hence these treatments by themselves have relatively little benefit for fruit and seed production (Oluwasanya et al., 2021a). BA functions to convert male flowers into female flowers, which leads to more fruits and seeds (Fu et al., 2014; Fröschle et al., 2017). When we combined pruning and BA, we obtained significantly more fruits and seeds than only pruning for 10 of 13 breeding progenitors. Thus, the combination of BA with pruning treatment, substantially increased the number of fruit and seed per plant, which is essential for generating sufficient progeny in crosses made in a breeding program.

The plant growth regulator STS, can improve inflorescence development, flower production, and flower longevity (Hyde et al., 2020; Oluwasanya et al., 2021a). However, in more than 70% of the genotypes in the present study, we did not see an increase in seed production when applying pruning and STS. This can be explained with similar reasoning as presented above for pruning. While STS increases total flower production, most of the flowers are male (Hyde et al., 2020; Oluwasanya et al., 2021a). When combining pruning and two growth regulators STS with BA, we observed a significant increase in the production of fruits and seeds, compared with pruning + STS (**Figure 4**). However, the numbers of fruits and seeds from two PGR, STS and BA, were similar to those from only one PGR, BA. The only exception is COL40 (**Figure 4**). STS treatment often results in leaf damage at high doses(Oluwasanya et al., 2021a). To minimize the toxic effect of STS, we applied STS through the leaf petiole, as suggested by Oluwasanya et al. (2021a). However, this procedure was difficult to implement in the field, where the wind would cause a disconnection between the petiole and the solution tube. Further development of the methodology might solve this problem. Considering the effect of pruning and growth regulators and the operational feasibility and efficiency in the field, we have tentatively concluded that the treatment of pruning and BA can be recommended for most cassava breeding applications.

When implementing the flower induction technology, we observed that, in approximately 5-10% of breeding progenitors, BA did not convert the male flowers into female flowers but a small fraction of them were hermaphroditic flowers with both male and female parts (Pineda et al., 2020b). The hermaphroditic flowers may be good for selfing or inbreeding. However, they might cause contamination when we want to do paired crosses, so emasculation or rouging of them may be required, which is potentially a slow process and take lots of effort. Studies have suggested that at higher doses of BA, the conversion to females is more complete (Oluwasanya et al., 2021a). Thus, we have established an ongoing test using a higher BA concentration to facilitate the complete conversion to female. On the other hand, in several breeding progenitors, BA affected the development of male flowers by reducing the amount of pollen produced. In turn, there was not enough pollen for crossing. To overcome this problem, we recommend that breeders leave several branches pruned but not sprayed with BA. The inflorescences from these branches will serve as the pollen providers, while the inflorescences with BA application provide female flowers for designed crosses.

### 4.2 Flower-inducing technology facilitates cassava speed breeding

Using the flower-inducing technology, we observed a more uniform flowering time among breeding progenitors. Most progenitors produced the first branches or flowers 2-4 months after planting under photoperiod extension. Plus, given the time required for the implementation of flower-inducing technology, pollination, and maturity of the seeds, we should be able to finish the pollination season within eight months. We observed that cassava seeds require three months to mature, accounting for about 40% of the seed production season. Most of the effort has been spent on flower-inducing, however, little attention has been given to promoting seed maturity. Seed biology study merits attention as well such that it will be enhanced to shorten the duration of seed maturity but maintain a good germination rate. Combining flower inducing and seed maturity studies, our target is to run crossing nurseries every 6 months, i.e., two pollination cycles per year. After we establish and validate the aggressive genomic selection protocol, where we cycle back the F_1_ clones to the crossing nursery, the duration of the breeding cycle will be reduced to 6 months. Compared with the normal 12-month crossing season, the genetic gains will be nearly doubled.

Regarding plant architecture which is linked to flowering time, cassava breeders have the dilemma that farmers generally prefer erect plant architecture with late flowering, but breeders need to make early flowering to shorten the pollination season and have rapid cycling. The flower-inducing technologies evaluated in this study provide a viable solution to the dilemma. We need to consider at least four factors when dealing with the dilemma. First, the location of crossing nurseries should be ~1,000 meters above sea level, to provide lower ambient temperature. Genotypes that grow erect in warmer low-elevation regions might be branching in cooler high-elevation growing regions. Second, photoperiod extension is more effective in a cooler location. Earlier flowering of erect breeding progenitors makes it possible to cross the farmer-preferred cultivars and generate new elite breeding populations. However, the early flowering progenitors do not show a decrease in the flowering time under photoperiod extension, so they can be planted under natural light. Third, little is known about the genetics of cassava flowering and related plant architecture. We have observed that several early-branching progenitors produced progeny with late branching, but systematic genetic mapping should be conducted to identify the major QTL and understand their dominant, codominant, or recessive inheritance of the favorable alleles. Then we can better design breeding schemes and paired crosses to shorten the pollination cycle and produce progeny with farmer-preferred erect plant architecture. Thus, by establishing crossing nurseries in a cooler location, extending the photoperiod, and enhancing genetic studies of plant architecture, breeders will solve the dilemma in cassava plant architecture soon and develop farmer-preferred cassava cultivars.

## Data availability statement

The raw data supporting the conclusions of this article will be made available by the authors, without undue reservation.

## Author contributions

EB carried out the experiments and collected the data; NM and SS managed the trials and supervised the operation; PH and TS provided the protocols, enhanced discussions and revised the manuscript; PK interpreted the results and enhanced discussions, JA analyzed data and interpreted results; XZ designed experiments, analyzed data and wrote the manuscript. All authors contributed to the article and approved the submitted version.
